# Intra-Articular Hyaluronic Acid for Knee Osteoarthritis: A Systematic Umbrella Review

**DOI:** 10.3390/jcm14041272

**Published:** 2025-02-14

**Authors:** Wojciech Michał Glinkowski, Wiesław Tomaszewski

**Affiliations:** 1 Center of Excellence “TeleOrto” for Telediagnostics and Treatment of Disorders and Injuries of the Locomotor System, Department of Medical Informatics and Telemedicine, Medical University of Warsaw, 02-091 Warsaw, Poland; 2 Stichting Med Partners, 1098 XH Amsterdam, The Netherlands; 3 Ars Medica Foundation for Medical Education, Health Promotion, Art and Culture, 03-301 Warsaw, Poland; 4 College of Physiotherapy, 50-038 Wrocław, Poland

**Keywords:** osteoarthritis, hyaluronic acid, viscosupplementation, intra-articular, molecular weight, non-surgical treatment, recommendations, guidelines

## Abstract

**Objective:** to evaluate the efficacy, safety, and cost-effectiveness of intra-articular hyaluronic acid (IAHA) in treating osteoarthritis (OA), considering innovations in formulations, comparative outcomes, and variability in guidelines. This review aims to synthesize evidence supporting the role of IAHA in multimodal treatment strategies. **Materials and Methods:** A general, narrative, umbrella review of systematic reviews and meta-analyses was conducted. Clinical practice recommendations and guidelines for IAHA use were also reviewed and evaluated. A comprehensive search was conducted across the main medical data sources. Inclusion criteria focused on studies evaluating the efficacy, safety, and impact of IAHA. Key outcomes included pain reduction (e.g., WOMAC, VAS), functional improvement, safety, and cost-effectiveness. **Results:** IAHA showed moderate efficacy in pain relief and functional improvement, especially in early-to-moderate OA. The results indicate that hybrid formulations and combination therapies show better clinical outcomes, with expanded efficacy and potential chondroprotection. However, heterogeneity between studies was noted, reflecting variability in patient populations and intervention protocols. International guidelines varied significantly, with some opposing routine use (e.g., AAOS, NICE) and others endorsing IAHA more or less conditionally (e.g., ESCEO, OARSI). **Conclusions:** IAHA remains a treatment modality in the arsenal of selected populations of people with OA, especially for early and moderate disease. High-quality, standardized studies are still needed to refine IAHA’s role and establish personalized guidelines for individual patients. A concerted effort to harmonize global recommendations and economic strategies, such as tiered pricing, can increase equitable access and optimize IAHA’s integration of multimodal treatment for OA.

## 1. Introduction

Osteoarthritis (OA) is a multifactorial degenerative joint disease characterized by the progressive degradation of articular cartilage, the remodeling of subchondral bone, synovitis, and osteophyte formation. It results from an imbalance between anabolic and catabolic processes in the joint microenvironment, driven by factors such as mechanical stress, aging, metabolic deregulation, and genetic predisposition. Osteoarthritis (OA) affects more than 7.6% of the global population and poses a significant socioeconomic burden. Intra-articular hyaluronic acid (IAHA) has emerged as a pivotal non-surgical intervention for alleviating symptoms and delaying disease progression [1,2]. Osteoarthritis (OA) is a chronic degenerative joint disorder that affects 7.6% of the global population, particularly the elderly, causing disability and impairing quality of life [1,2,3,4,5,6,7,8]. The rising prevalence of OA due to aging populations, obesity, and reduced physical activity poses a substantial burden on individuals and healthcare systems, primarily affecting weight-bearing joints such as the knees and hips [3,4,5,6]. The socioeconomic impact of OA encompasses direct healthcare costs, productivity losses, and disability-related expenses, with low- and middle-income countries bearing a disproportionate burden owing to limited access to diagnostics and treatments [6,7]. Comprehensive multidisciplinary management, early interventions, lifestyle changes, and innovative treatments are crucial for reducing the socioeconomic burden of OA [8,9,10,11]. OA cases have surged by 132.2% since 1990, with a significant increase expected by 2050 across various joints [8,9,10,11]. The growing OA burden is attributed to increasing life expectancy and obesity rates, leading to higher economic costs and reduced quality of life [3,6,12,13,14]. Despite advancements in understanding osteoarthritis (OA) pathophysiology, effective management remains challenging. Current therapies focus primarily on symptom relief without addressing the underlying mechanisms of joint degeneration. The lack of disease-modifying OA drugs and variability in patient responses further complicate efforts to halt disease progression [3,8,12,15,16,17]. IAHA is considered a treatment for OA with a biological and clinical rationale, including replenishing hyaluronic acid in the synovial fluid, which restores joint homeostasis with anti-inflammatory, chondroprotective [1,18], and analgesic effects [8]. However, IAHA adoption faces challenges due to disparities in access, patient expectations, and combination therapies. Despite numerous treatments, there is a need for more effective long-term strategies to relieve symptoms and delay surgical interventions. Hyaluronic acid (HA), a glycosaminoglycan in synovial fluid, maintains joint homeostasis [19,20]. HA’s viscoelastic properties are crucial for joint lubrication, shock absorption, and smooth movement [21,22,23,24,25,26]. Viscosupplementation (VS) with IAHA has become essential [27,28,29,30,31,32,33,34,35,36,37,38,39,40] and involves direct injection into the joint [37,38,41,42,43,44,45,46,47,48,49,50,51,52,53]. IAHA restores the viscoelastic properties of synovial fluid, reduces pain, and improves joint function, potentially delaying surgeries such as total joint replacement [54,55,56,57,58,59]. IAHA improves lubrication and shock absorption by replenishing the synovial fluid [22,31,60,61,62,63]. Its role in OA management is controversial owing to inconsistent global Clinical Practice Guidelines (CPGs), varying study designs, patient populations, and outcome measures, leading to polarized clinician views. High costs, limited insurance coverage, geographic disparities, and patient misconceptions regarding IAHA efficacy have hindered its real-world adoption [64]. Harmonized guidelines and strategies are required to optimize the use of IAHA. Innovations such as hybrid HA formulations, physical therapy, platelet-rich plasma (PRP), weight loss programs, and biomarker-based patient selection aim to improve efficacy, durability, and cost-effectiveness. The literature observes variability in IAHA recommendations in international guidelines [15]. Over the years, changes in opportunities for optimization through innovative formulations, combination therapies, and personalized medicine have become evident. Published papers have also identified barriers to implementation in the real world and proposed practical solutions. IAHA manages OA through short-term symptom relief and long-term strategies [1,65]. While socioeconomic factors such as cost and insurance constraints significantly affect the availability and adoption of advanced IAHA therapies, this review focuses primarily on therapeutic mechanisms and clinical innovations. This study aimed to address inconsistencies in international guidelines and barriers to the adoption of intra-articular hyaluronic acid (IAHA) therapies, with a focus on optimizing use through innovations such as combination therapies and patient stratification [1,65].

## 2. Materials and Methods

This review utilized a systematic and narrative approach to critically evaluate the clinical utility of IAHA VS in OA management. This study adhered to the Preferred Reporting Items for Systematic Reviews and Meta-Analyses (PRISMA) guidelines for systematic reviews to ensure methodological rigor and transparency [66].

A comprehensive search was conducted across data sources, including PubMed, Cochrane Library, EMBASE, Scopus, and Web of Science, for studies published between 2014 and November 2024. The search strategy was based on keywords that included “hyaluronic acid”, “intraarticular hyaluronic acid”, “viscosupplementation”, “osteoarthritis”, “guidelines”, and “recommendations”. Additional data were extracted from the international guidelines, CPGs, and expert consensus statements. The literature search was designed to identify systematic reviews, meta-analyses, and narrative reviews relevant to the use of IAHA in OA. The search strategy excluded primary clinical trials and focused on secondary evidence sources. Key terms consisted of (“osteoarthritis” OR “knee osteoarthritis” OR “hip osteoarthritis”) AND (“hyaluronic acid” OR “IAHA” OR “intra-articular hyaluronic acid”) AND (“review” OR “systematic review” OR “meta-analysis” OR “narrative review”). The filters included the English language, reviews were set as the type of articles, and the publication date was set to include reviews published from 2014 to 2024.

### Inclusion Criteria

Meta-analyses, systematic reviews, and clinical guidelines were included. The included population covered adults with OA (knee, hip, or other significant joints) stratified by disease severity (early, moderate, or advanced). IAHA formulations, including hybrid complexes and combination therapies, were considered the intervention. The outcomes included efficacy, safety, adverse events, and economic evaluation. Among the outcome measures considered during the analysis were pain relief (WOMAC and VAS scores), functional improvement (WOMAC function), safety (adverse events and tolerability), long-term efficacy (3–12 months), cost-effectiveness, and delay of surgical intervention.

The PICO framework guided the literature review process. Population (P) encompassed adult patients with knee or hip osteoarthritis; Intervention (I) comprised intra-articular hyaluronic acid (IAHA), administered either as a monotherapy or in combination with other therapeutic modalities; Comparator (C) included placebo, corticosteroids, or alternative intra-articular injections; and Outcomes (O) focused on clinical endpoints such as pain alleviation, functional enhancement, safety profile, and cost-effectiveness.

Exclusion criteria consisted of RCTs, non-peer-reviewed studies, conference abstracts, pediatric populations, and non-OA diseases.

Two independent reviewers were responsible for data extraction and quality assessment, and reviewed the studies based on their design, patient demographics, OA severity, type of intervention, and comparator. The data synthesis was conducted using a simple spreadsheet for organizational purposes, and references were managed with EndNote 21.

Among the methods of analysis, descriptive statistics were used to summarize the characteristics of the study. Subgroup analyses are performed by molecular weight, disease severity, and regional guideline differences. Two independent reviewers screened the selected studies for eligibility and resolved disagreements by consensus. Extracted data included the study design, sample size, patient demographics, OA severity, IAHA formulation, dosing schedule, combination therapy, primary outcomes (pain relief, functional improvement, and adverse events), and secondary outcomes (economic evaluation and quality of life). The AMSTAR tool assessed the quality of included meta-analyses.

Descriptive statistics were used to summarize the characteristics of the study. Study heterogeneity was assessed via the I^2^ statistic. Subgroup analyses were used to evaluate the impact of IAHA molecular weight (low, medium, high, and hybrid), disease severity, patient phenotypes (age, obesity, and comorbidities), and geographic variations in guideline adherence. The parameters included study design, demographics, interventions, comparators, outcomes, central tendency measures, variability, proportions, and percentages.

## 3. Results

**Figure 1:**
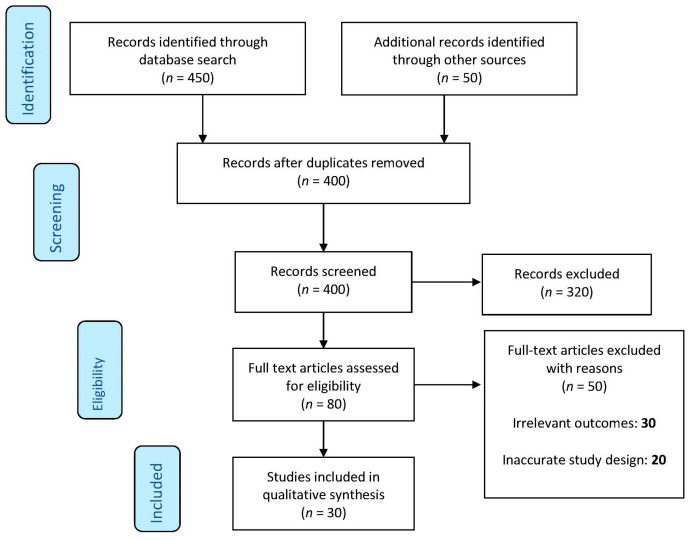
Flow chart of the literature search using the PRISMA (Preferred Reporting Items for Systematic Reviews and Meta-Analyses).

The study used a rigorous and transparent methodological approach to the PRISMA guidelines (Figure 1).

Descriptive statistics were used to summarize the study design proportions, population characteristics, and outcomes. Subgroup analyses focused on IAHA formulations and comparator treatments. The results were qualitatively synthesized using means and standard deviations for continuous variables and proportions for categorical data. A narrative approach was used to contextualize the findings within the variability of the included studies and methodologies. The results were tabulated and visualized to elucidate trends and to compare IAHA with other efficacy, safety, and cost-effectiveness treatments. Significant heterogeneity was observed in patient populations, comparators, and outcome measures, necessitating standardized protocols for future trials. The systematic reviews and meta-analyses extracted study characteristics, comparators, outcomes, adverse events, and heterogeneity. The selected references constitute the evidence base for the findings summarized in Table 1.

AMSTAR 2 was performed (Table 2) to provide a more detailed assessment of methodological quality for systematic reviews and meta-analyses, including evaluation of aspects like protocol registration, comprehensive search strategies, study inclusion, and assessment of heterogeneity.

Strengths included a well-documented search strategy that ensured comprehensive study inclusion and clear study inclusion criteria. However, quality assessment was not consistently reported. The extracted data included study designs, intervention types (e.g., high- or low-molecular-weight IAHA), and outcomes. Random-effects models are commonly used to account for heterogeneity and consistent data extraction, and synthesis increases comparability.

High heterogeneity in patient populations (e.g., severity of OA) and treatment regimens (e.g., IAHA molecular weight) was reported, making subgroup analyses difficult. Few meta-analyses disclosed funding sources or potential conflicts of interest that might have affected the results. Meta-analyses effectively combined the effect sizes for pain and functional outcomes using random-effects models to account for heterogeneity. However, the temporal endpoints (e.g., six vs. twelve months) have not been consistently reported.

The I^2^ statistic was used to measure the percentage of variability between studies due to heterogeneity rather than chance. Determining whether the combined results of systematic reviews and meta-analyses were reliable was crucial. Meta-analyses combining IAHA with comparator drugs such as PRP and corticosteroids have reported I^2^ values exceeding 50%, indicating moderate-to-significant heterogeneity. This reflects the differences in the study design and population characteristics. IAHA safety had lower I^2^ values (less than 30%) in the safety domain, suggesting low heterogeneity, probably because of the consistent reporting of adverse events across studies. In the cost-effectiveness domain and combination therapies, heterogeneity was high (>75%) in studies evaluating cost-effectiveness and IAHA combinations (e.g., IAHA + PRP), reflecting different methodologies and differences in health systems.

The analyzed publications covered clinical issues related to intra-articular hyaluronic acid (IAHA) use, such as clinical and long-term efficacy, repeated injections, safety, guideline variability, innovations in formulations, combination therapies, economic and real-world factors, patient phenotypes, personalized medicine, and comparative efficacy with emerging regenerative therapies in osteoarthritis (OA) management. A literature review revealed that IAHA consistently alleviates pain and improves joint function in patients with knee osteoarthritis (OA). Across randomized controlled trials (RCTs) and meta-analyses, IAHA was associated with statistically significant reductions in pain and improvements in functional scores, mainly when assessed using the Western Ontario and McMaster Universities Osteoarthritis Index (WOMAC) and Visual Analog Scale (VAS). High-molecular-weight and cross-linked formulations demonstrated greater efficacy and durability than low-molecular-weight preparations, with symptom relief lasting up to six months post-injection [1,15,39,89,90,91]. However, the magnitude of these benefits varies across the studies. Meta-analyses reported effect sizes ranging from 0.30 to 0.40 compared to placebo, which is clinically meaningful but modest.

A comparison of the efficacy of IAHA and new regenerative therapies in the treatment of OA has shown a key role in the non-surgical treatment of OA, especially KOA. Many studies have shown that IAHA significantly reduces pain and improves function within 4–6 weeks, with effects lasting up to six months. However, the clinical efficacy of IAHA is often questioned using PRP and Bone Marrow Aspirate Concentrate (BMAC) injections, and recent studies have shown long-term benefits in patients with KOA [21].

Comparisons with other injectables, such as corticosteroids, highlighted IAHA’s slow-onset but prolonged effect, whereas PRP showed potentially more significant long-term benefits [2,74,75]. Table 3 summarizes the impact of delivery therapies on their efficacy, safety, and cost-effectiveness.

Studies have increasingly highlighted the importance of combination therapies that show the synergistic effects of IAHA in combination with PRP, which has shown better long-term benefits in pain reduction and functional improvement than IAHA alone. Combining PRP with IAHA can enhance chondroprotective effects [2,92]. The intra-articular effects of PRP were compared with those of IAHA. PRP has demonstrated superior long-term outcomes in pain reduction and functional improvement compared to intra-articular hyaluronic acid (IAHA) alone. Studies have shown that PRP’s regenerative properties, which stimulate cartilage repair and modulate inflammation, result in sustained benefits lasting over 6–12 months, whereas IAHA typically provides symptom relief for 3–6 months. The enhanced effects and synergy of PRP and IAHA combinations have been observed for joint preservation. This has shown additive effects by leveraging IAHA’s viscoelastic support of IAHA with PRP’s regenerative potential of PRP. Combining both improves cartilage health, reduces inflammation, and offers immediate and extended relief from symptoms. Similarly, studies suggest that PRP and IAHA combinations enhance matrix synthesis, reduce cartilage degradation, and improve joint functionality compared to either treatment alone. A review of 38 randomized controlled trials involving 5025 patients revealed IAHA’s significant pain and functional relief, although its effects diminish over time compared to newer treatments. Meta-analyses suggest that PRP and BMAC injections outperform IAHA in critical outcomes, such as WOMAC and VAS scores [93,94]. PRP offers superior relief [35,46,93,94,95,96,97,98,99,100,101,102,103,104,105,106,107,108]. IAHA efficacy, which is dependent on molecular weight, provides joint lubrication but lacks PRP’s regenerative potential of PRP, which supports cartilage repair through growth factors [87,88]. Studies have consistently shown the some inferiority of IAHA to PRP [35,46,93,94,95,96,99,102,104,106,107,109,110] and BMAC [74,93]. Belk et al. [93] found that PRP significantly improved pain, function, and quality of life beyond six months, unlike IAHA’s transient benefits. Raeissadat et al. [108] confirmed PRP’s superiority in a one-year KOA trial. BMACs promote tissue regeneration and offer more durable benefits than IAHA in long-term OA management [94]. Reviews have suggested that PRP injections yield better long-term outcomes than IAHA injections, particularly in pain and function [93]. IAHA’s benefits last six months, whereas PRP and BMAC provide sustained relief and potential tissue regeneration [93,94,108]. Zhao et al. [35] showed that adipose-derived mesenchymal stem cells (MSCs) and leukocyte-poor PRP outperform IAHA at six and twelve months, with MSCs providing the most significant pain relief (WMD −20.93 at twelve months). Cao et al. [111] demonstrated superior pain relief and functional improvement in MSC therapies compared to IAHA. This evidence supports the preference for PRP and BMAC, challenging IAHA’s continued use. Future research should compare these treatments directly and consider combining IAHA with regenerative therapies to enhance outcomes [21,93,108].

Combining IAHA with physical therapy and structured rehabilitation programs yielded enhanced joint mobility and reduced stiffness, particularly in early-stage OA [2,31,112,113]. Lifestyle modifications, including weight loss and activity modification, amplify IAHA’s benefits, especially in overweight and obese populations [1,91].

In terms of long-term efficacy and repeated injections, IAHA provides moderate pain relief and improved joint function in KOA disease for 3–12 months, with optimal results within the first 6 months [21,84,114,115,116]. However, its long-term superiority over placebo and other treatments remains inconclusive owing to heterogeneous study designs, HA formulations, dosing regimens, and patient populations [67,94,104]. Despite favorable safety profiles with minor transient side effects [21,84,114,115,116], the lack of standardized protocols and rare long-term follow-ups beyond six months limits the quality of evidence [67]. IAHA injections, potentially possessing senomorphic properties [116], should be part of an individualized, comprehensive treatment plan for KOA and ankle OA [42,96]. Future research should prioritize standardized methodologies and extended follow-ups to clarify HA’s enduring benefits of IAHA and optimize therapy protocols. Viscosupplementation demonstrates substantial long-term efficacy in managing OA with repeated injections, offers sustained symptom control, and delays invasive procedures. Future studies should compare HA’s long-term efficacy with other treatments, optimize dosing schedules, and repeat injection protocols for better outcomes. However, DeRogatis et al. [117] emphasized the inconsistent long-term outcomes of IAHA, with studies often reporting short-term pain relief, typically not exceeding six months.

IAHA has shown a favorable safety profile with transient and self-limiting local adverse events, such as mild pain or swelling at the injection site, from a safety and tolerability perspective. Systemic side effects are rare, with no serious adverse events consistently associated with IAHA, making it safer than oral NSAIDs and corticosteroids in terms of gastrointestinal, cardiovascular, and metabolic risks [1,92]. The safety of IAHA injections [23,27,30,31,34,36,62,79,81,85,118,119,120,121,122] was consistent, showing mild and transient adverse events, such as injection site pain, swelling, and stiffness in less than 10% of patients, with no severe systemic adverse effects reported. Compared with corticosteroids and other intra-articular treatments, IAHA has a significantly lower rate of adverse effects, reinforcing its safety for OA treatment [49,103]. Adverse events are rare but can occur in the form of severe acute local reactions (SALRs) at the onset of acute joint pain, associated swelling, erythema, and restriction of movement after intra-articular injection of hyaluronic acid. The onset of symptoms ranged from 4 h to several days. SALR appears to be an immune reaction to HA and is associated with improper injection techniques. The risk of complications associated with such injections is low, and mild and severe SALRs occur. These reactions can be mitigated by carefully selecting the injection site and using ultrasound navigation [123].

The medical community has debated the use of IAHA for OA. This literature review synthesizes current guidelines and recommendations for IAHA use based on recent high-quality systematic reviews and clinical practice guidelines. Clinical guidelines and recommendations from major medical organizations on using IAHA in OA and recommendations for VS articles were analyzed and deemed appropriate for inclusion. The articles included systematic reviews, procedures, and cross-sectional analyses with a high-to-moderate level of evidence.

The differing clinical guidelines on HA use complicate decision making. Some recommend HA as part of a multimodal treatment for OA, while others question its efficacy compared to alternatives such as corticosteroids or PRP. PRP offers long-term pain relief and functional improvement, but UHMW HA is cost-effective and reduces the injection frequency. Despite limited evidence, several high-quality guidelines have consistently recommended IAHA for OA [124]. Nonetheless, there is little consensus on its use, with some guidelines supporting it under specific conditions or as part of a comprehensive treatment plan that includes exercise, patient education, weight management, and NSAIDs [2,124]. Various orthopedic and rheumatologic associations include IAHAs in KOA treatment when nonpharmacological and primary pharmacological therapies fail. HA injections aim to restore the viscoelastic properties of synovial fluid, relieve pain, improve joint function, and potentially delay surgeries such as total joint arthroplasty (TJA). Despite its widespread use, HA’s efficacy is debated, with guidelines varying based on available evidence. The IAHA application guidelines and recommendations are based on updated systematic reviews and clinical practice guidelines.

1. The Osteoarthritis Research Society International (OARSI) conditionally recommends IAHA for KOA, especially for patients who do not respond to other treatments such as NSAIDs or physical therapy. IAHA is recommended as part of a broader multimodal treatment approach, particularly when first-line therapies fail [69]. The Osteoarthritis Research Society International (OARSI) [69,125,126] maintains the IAHA recommendations as preliminary, emphasizing the need for further solid evidence. OARSI recognizes the heterogeneity of clinical responses based on patient phenotypes and disease stages [18,65].
2. The European Society for Clinical and Economic Aspects of Osteoporosis, Osteoarthritis, and Musculoskeletal Diseases (ESCEO) supports IAHA as a second-line treatment for KOA, mainly when NSAIDs are ineffective or contraindicated [125,127]. IAHA is emphasized for pain relief and functional improvement in OA’s early-to-moderate stages [127]. The European Society for Clinical and Economic Aspects of Osteoporosis and Osteoarthritis (ESCEO) advocates for IAHA as a core part of OA management, particularly in early-to-moderate disease stages. ESCEO endorses IAHA because of its dual benefits of symptom relief and potential chondroprotection [1].
3. Due to inconsistent evidence of its benefits, the American Academy of Orthopaedic Surgeons (AAOS) strongly advises against using IAHA for KOA and HOA [15,89,101,128]. However, IAHA may be appropriate for specific patient populations that do not respond to other treatments [15]. The American Academy of Orthopaedic Surgeons (AAOS) does not recommend the routine use of IAHA due to limited evidence for significant clinical benefits. This position is primarily based on high-stringency thresholds for clinical significance and preference for outcomes with large effect sizes (e.g., greater than 0.5 WOMAC improvement) [15,65,86].
4. The American College of Rheumatology (ACR) and Arthritis Foundation (A.F.) conditionally recommend using IAHA for KOA, citing mixed evidence of its efficacy. Although IAHA may offer benefits, it is often modest and inconsistent, making it less favorable than other corticosteroid treatments [129]. The American College of Rheumatology (ACR) provides conditional recommendations for IAHA use in select patients, particularly those who fail to respond to other non-surgical options such as NSAIDs and physical therapy. The ACR emphasizes individualized decision making based on patient-specific factors [2,92,112,129,130,131].
5. The International Cartilage Regeneration and Joint Preservation Society (ICRS) does not explicitly endorse intra-articular hyaluronic acid (IAHA) for early and moderate osteoarthritis (OA) with specific claims of maintaining joint health, slowing cartilage degradation, and delaying surgical interventions [132].
6. The International Symposium on Intra-Articular Treatment (ISIAT) [18,132] recommends IAHA for mild-to-moderate KOA, highlighting innovative products that significantly and sustainably improve pain, joint function, and quality of life. The focus is on developing IAHA products that outperform the current viscosupplements. ISIAT emphasizes addressing the clinical needs of KOA treatment more effectively. The efficacy and safety of IAHA are its key benefits. Despite some debate regarding the extent of its benefits, IAHA is noted for significant pain relief and functional improvement in many patients with KOA, especially within a comprehensive treatment plan. The ISIAT stresses the need to align guidelines from other organizations to standardize IAHA features and clinical endpoints, thereby enhancing patient care [102,132]. The ISIAT emphasizes the need for further research on patient selection criteria and treatment protocols to tailor IAHA treatment to individual patients and to optimize outcomes [121].
7. The European League Against Rheumatism (EULAR) provides general guidance on intra-articular therapies but does not explicitly endorse or reject IAHA for KOA. These guidelines emphasize the importance of individualized care and patient selection, noting the variability in treatment responses [127].
8. The American Medical Society for Sports Medicine (AMSSM) supports using IAHA in managing KOA, particularly in athletes and physically active individuals. IAHA is highlighted for managing OA symptoms and maintaining joint function, emphasizing the need for more invasive procedures such as surgery [133].
9. The Italian Society of Orthopaedic Surgery (SIOT) supports using IAHA, especially in combination with other treatments, such as PRP. IAHA is favored for its long-term safety profile and potential benefits in delaying surgical intervention [134].
10. The French Rheumatology Society recommends IAHA for KOA, particularly for symptom relief, without expecting chondroprotective effects. IAHA is appropriate in patients with symptomatic KOA and minimal effusion [135].
11. The Chinese Guidelines for Osteoarthritis recommend IAHA for KOA with persistent or moderate-to-severe pain, suggesting its use to improve symptoms and delay joint replacement surgery [136].
12. The South African Rheumatism and Arthritis Association (SARAA) supports VS, particularly UHMW HA formulations, for KOA in patients requiring sustained relief and aiming to delay surgical interventions [137].
13. The Brazilian Society of Orthopaedics and Traumatology (SBOT) advocates using UHMW HAs for extended joint lubrication and pain relief in patients with OA [10].
14. Korean recommendation guidelines support the conditional use of IAHA for joint symptom control when glucocorticoid injections or other interventions fail [138].
15. The Spanish Society of Rheumatology recommends VS as an adjunct treatment for OA, favoring UHMW HAs for their potential to offer longer-lasting pain relief and improved quality of life [139].
16. The Indian Rheumatology Association supports IAHA for OA management and recommends UHMW HA formulations for enhanced viscoelastic properties and sustained symptom relief [140].
17. The Swiss Society of Rheumatology has expressed caution regarding routine IAHA use, highlighting the need for individualized patient assessment and more comprehensive evidence regarding long-term efficacy and safety [141].
18. The German Orthopaedic Society (DGOU) recommends cautious recommendations for IAHA, indicating potential benefits but emphasizing the need for more robust clinical trials to establish efficacy [142].
19. The National Institute for Health and Care Excellence (NICE) does not recommend VS as a routine treatment for OA [143] due to a lack of apparent efficacy and cost-effectiveness data.
20. The EUROVISCO Group provides detailed recommendations for clinical trials to assess the disease-modifying effects of IAHA in KOA patients. This group emphasizes the need for standardized study designs, including imaging and biological markers, to optimize trial outcomes [39,90,91].

Several societies/groups believe that IAHA benefits include pain relief, improved joint function, and delayed surgical intervention. At the same time, limitations include variable effectiveness, limited long-term benefits, and cost-effectiveness concerns. Recommendations vary; some organizations conditionally support IAHA based on patient profiles or disease stages, whereas others discourage its routine use. The inconsistency in international guidelines for HA viscosupplementation reflects mixed evidence regarding its efficacy in OA management. Although some studies have reported significant short-term benefits, alternative therapies’ long-term effectiveness and comparative outcomes remain unclear. This inconsistency necessitates further high-quality research to establish clinical guidelines and optimize HA treatment protocols for OA management. Healthcare systems, economic factors, and regulatory policies influence global disparities and geographical differences in guideline adherence. For example, European countries have higher IAHA adoption rates than North America, where insurance coverage limitations significantly restrict its use [1,31,113]. Guidelines and recommendations from key medical organizations, both internationally and in some countries, regarding using IAHA in treating OA show considerable variability [2,69,90,121,124,125,126,129,131,134,144,145,146]. Some guidelines recommend HA as a second-line treatment after NSAIDs or when first-line therapies are contraindicated. At the same time, others have expressed caution owing to inconsistent clinical trial evidence, particularly regarding long-term efficacy [89,126]. This discrepancy underscores the need for standardized protocols and further research to clarify IAHA’s role in OA treatment [27]. Although the clinical efficacy of IAHA in OA treatment has been extensively studied, findings have been inconsistent. IAHA provides significant pain relief and functional improvement at various time points post-injection; however, its efficacy varies according to product and study design, contributing to inconsistent recommendations. Compared to intra-articular corticosteroids (IACSs), IAHA shows comparable short-term efficacy and longer-lasting effects. IACSs offer immediate pain relief, typically lasting four weeks, whereas IAHA shows a more durable response. These guideline differences impact clinical decision making, especially when choosing treatments for specific patient populations. The use of IAHA has become routine, but the availability of new tests has significantly decreased. Standardizing recommendations remains challenging, and further clinical trials are necessary. The lack of high-quality, head-to-head studies comparing IAHA with treatments such as PRP or mesenchymal stem cells (MSCs) complicates the standardization of clinical recommendations. Variability in trial methodologies and patient populations further complicates the generation of uniform guidelines, as reflected in Cochrane reviews [27]. These evidence-based discrepancies contribute to the differing international guidelines. Some regions promote IAHA as a viable OA treatment, while others suggest its limited use owing to inconclusive evidence on long-term benefits or optimal dosing regimens [27]. Some guidelines support IAHA use under specific conditions or as part of a broader treatment strategy, including exercise, patient education, weight management, and NSAIDs [2,124]. Many guidelines and clinical recommendations developed by specialty organizations, such as orthopedics and rheumatology, consider the IAHA part of a comprehensive treatment plan for OA when nonpharmacological and essential pharmacological treatments fail. Table 4 summarizes the recommendations of the selected associations over the past decade.

Opinions on IAHA use differ. Some recommend delaying surgery for early and moderate OA, whereas others question the inconsistent evidence and cost-effectiveness. Most guidelines customize treatment based on the patient’s needs and disease progression. Bichsel et al. [147] identified inconsistencies in the recommendations for IAHA use. Their guidelines support a multimodal approach emphasizing patient education and exercise [147]. Professional societies vary in endorsing IAHA, indicating a lack of consensus regarding its efficacy and timing. These findings suggest that IAHA could be beneficial in treating delayed surgeries such as arthroplasty.

## 4. Discussion

Recommendations and guidelines often cite numerous mechanisms raised to justify the effect of IAHA, which exhibits anti-inflammatory properties, modulates local immune response [148,149], stimulates endogenous IAHA production [31,150], and promotes joint homeostasis [9,31,48,151]. IAHA enhances synovial fluid viscoelasticity, improves shock absorption and lubrication [24,152,153,154], reduces pro-inflammatory cytokines and mediators [155,156,157], and supports cartilage matrix maintenance and repair [158,159,160]. IAHA preserves subchondral bone integrity by promoting osteoblast activity, suppressing osteoclastogenesis, and modulating Wnt/β-catenin signaling [68,161,162,163,164,165]. It maintains synovial membrane and cartilage matrix integrity through various mechanisms [22,166,167,168], enhances proteoglycan retention, forms essential aggregates with aggrecan, and conserves ECM proteoglycans [19,22,35,87,98,100,101,102,103,104,105,106]. IAHA mechanisms collectively improve therapeutic outcomes and patient quality of life in OA management [27,29,31,32,56,78,91,120,121,122,169,170], with effectiveness varying based on molecular weight, disease severity, and patient characteristics [21,23,26,120]. While IAHA injections have shown symptomatic relief and enhanced joint functionality [21,28,32,43,44,51,112,118,119,120,121,122,123,124], the extent of HA’s benefits and comparative effectiveness remain topics of discussion [21,22,23,56,118,171,172].

A critical analysis of the controversy indicates that IAHA delivery injections have been widely studied for OA, and clinical trials have often confirmed their efficacy and safety [32,70,113]. However, their inclusion in clinical guidelines varies with organizations, such as AAOS-expressing reservations [15,89,101,128]. The inconsistency in recommendations stems from differing evidence thresholds, methodological interpretations, real-world applicability, and healthcare priorities. Clinical studies have highlighted the many critical benefits of IAHA.

The latest developments and innovations in IAHA formulations, including hybrid cooperative complexes combining high- and low-molecular-weight hyaluronic acids, have improved clinical outcomes [21,47,55,61,92,173,174,175]. Hybrid formulations better replicate the natural viscoelasticity of synovial fluids; improve joint lubrication, shock absorption, and chondroprotection; and offer extended duration of efficacy with fewer injections [47,92,149,171,174,176,177]. Hybrid complexes provide longer-lasting symptomatic relief, reduce the injection frequency, and improve patient adherence. By addressing biomechanical and inflammatory aspects, these advanced formulations are particularly effective in managing symptoms in patients with moderate-to-severe osteoarthritis (OA), where traditional therapies may fall short [2,92,130]. Emerging evidence supports integrating these innovative products into standard OA care, particularly for patients with moderate-to-severe disease.

Economic and real-world evaluations underscore IAHA’s cost-effectiveness in delaying surgical interventions such as total knee replacement [58,130,178]. However, accessibility challenges persist, especially in low-resource settings where reimbursement policies vary widely [55,125,127]. Educating patients about realistic IAHA performance expectations is key to improving adherence to and satisfaction with treatment [18,92].

A promoted approach for treating patients is to focus on patient phenotypes and personalized medicine. Stratification by patient characteristics revealed variability in IAHA efficacy and showed that patients with early-to-moderate OA, a low BMI, and minimal inflammatory markers benefited the most. Patients who do not respond or respond poorly to IAHA have advanced osteoarthritis, severe deformities, and significant comorbidities that reduce the effectiveness of IAHA [1,92]. Personalized approaches using biomarkers to predict response to IAHA are emerging as promising avenues for optimizing treatment outcomes [1,91]. IAHA plays a valuable role in OA management, particularly when tailored to patient-specific characteristics and integrated into multimodal strategies. Despite its proven efficacy and safety [31,70,79,85,177], guideline inconsistencies and real-world barriers highlight the need for harmonized recommendations and continued research to refine its clinical application [1,39,49,121,124,127,143,145,179].

Numerous RCTs have demonstrated significant pain reduction [30,32,127,146,147], as measured by the WOMAC pain scale and VAS, especially in early-to-moderate OA lasting up to six months, indicating efficacy in symptom relief and reduction. IAHA also enhances joint function and mobility and reduces stiffness [32,86] as a non-surgical option to alleviate symptoms and delay surgeries, such as TKA [58,130,178]. Compared with corticosteroids, IAHA offers a slower, longer-lasting effect and immediate relief, making it suitable for chronic treatment [2,53,100,105,145]. IAHA’s favorable safety profile stems from typically mild, transient local reactions (e.g., injection site pain) and avoidance of systemic risks associated with oral NSAIDs or corticosteroids, such as gastrointestinal and cardiovascular side effects [31,36,81,85]. It benefits patients contraindicated for NSAIDs, those with delayed surgery, and those with early-stage OA.

However, guidelines remain cautious owing to stringent evidence thresholds, study design heterogeneity, high placebo effect, focus on population-level applicability, and limited evidence of long-term benefits. ESCEO recommends IAHA as a second-line treatment for early-to-moderate OA. OARSI conditionally recommends it, acknowledging the variability in efficacy. Conversely, the ACR conditionally recommends IAHA for patients who have failed other nonsurgical options, emphasizing individual decision making. Real-world factors influence IAHA recommendations, including cost-effectiveness, local politics, and rehabilitation policies. IAHA is more expensive than nonsteroidal anti-inflammatory drugs (NSAIDs) and corticosteroids. According to AAOS, treatment benefits often do not justify costs. Limited reimbursement policies in the United States restrict access and reduce the perceived value. NICE strongly opposes routine IAHA use owing to its insufficient efficacy and cost-effectiveness. Geographic and systemic differences influence IAHA use in Europe and are often adopted directly. This is not always reflected in guidelines such as ESCEO skepticism, as seen in North American organizations such as AAOS and NICE.

To increase evidence credibility, aligning clinical trial support for IAHA with guideline skepticism requires standardized study designs, patient selection criteria, and comparison arms. Developing biomarkers and predictive markers for the IAHA response can target subgroups likely to benefit. Long-term studies evaluating repeated injections and their outcomes must address sustained efficacy and global collaboration concerns. Harmonized guidelines agreed upon by specialty organizations can reduce discrepancies and provide precise recommendations. Independent scientific associations require external studies to reach a consensus.

Clinical studies have demonstrated IAHA’s effectiveness in pain relief and improved function and safety in specific OA subgroups. However, guidelines such as AAOS remain cautious owing to stringent efficacy criteria, heterogeneous evidence, and practical issues, including cost and placebo effects. Standardized trial protocols, individualized patient recommendations, and comprehensive longitudinal data are required to clarify IAHA’s role in the treatment of OA. Methodological inconsistencies in IAHA meta-analyses lead to variable conclusions regarding efficacy and safety, complicating guideline consistency. Including diverse patient groups and OA severity levels in meta-analyses may weaken the efficacy results because IAHA is less effective for advanced OA. Variations in the participants’ baseline characteristics (age, BMI, and comorbidities) affected the outcome variation. Different assessment metrics and instruments (WOMAC, VAS, and KOOS) complicate the data aggregation. Internationally uniform PRO assessments can standardize results and influence the recommendation consensus. Discrepancies in study follow-up durations (short-term ≤6 months vs. extended follow-up) led to inconsistent assessments of IAHA’s sustained effects. Meta-analyses often aggregate studies that compare IAHA with different interventions (placebo, corticosteroids, and NSAIDs), obscuring direct efficacy comparisons. Inadequate control of strong placebo responses during delivery may overestimate IAHA’s therapeutic effects.

Differences in IAHA formulations, such as molecular weight, cross-linking, and administration protocols, significantly affect product efficacy. Meta-analyses often fail to account for these variations, potentially overestimating efficacy due to publication bias and favoring positive results. Integrating IAHA into personalized treatment paradigms for OA should involve tailored regimens based on individual patient profiles, reflecting OA’s multifaceted etiology of OA, including mechanical, inflammatory, and lifestyle factors.

IAHA’s viscoelastic and chondroprotective properties complement physical therapy and weight control, countering mechanical stress and inflammation. Combining IAHA with PRP or corticosteroids offers short-term relief and long-term joint protection through synergistic effects. Biomarkers and phenotypic profiling can identify patients likely to respond to IAHA, such as those with early-to-moderate OA or low synovitis levels, thereby optimizing personalized therapy.

**Scheme 1:**
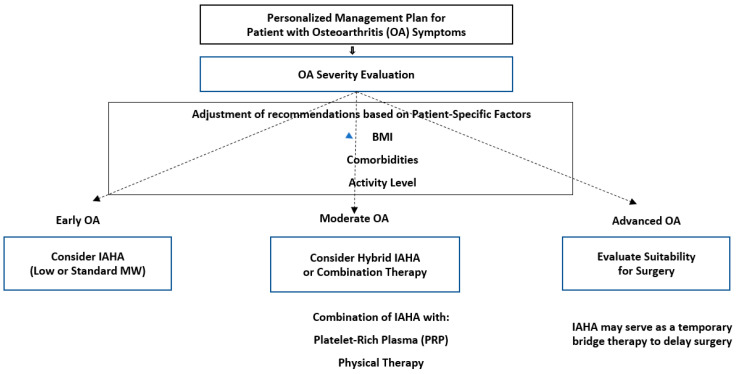
Treatment decision making based on patient phenotypes in IAHA.

Publications suggest that combining IAHA with structured exercise and lifestyle modifications can delay surgical interventions, such as total joint arthroplasty, especially in younger patients. Individualizing treatment goals based on OA severity, comorbidities, and activity levels maximize therapeutic benefits. High-molecular-weight IAHAs can be prioritized for long-term symptom relief, whereas low-molecular-weight formulations are suitable for rehabilitation protocols. Personalized plans tailored to expectations and based on a patient’s phenotype can improve adherence and treatment satisfaction (Scheme 1).

Economic solutions, such as tiered pricing and broader health insurance coverage, can enhance cost-effectiveness and promote equitable access to IAHA treatment [2,18].

Tiered pricing allows IAHA to offer products at variable prices according to health system resources, geographic regions, or patient income, making IAHA affordable in low- and middle-income countries while remaining profitable in high-income areas and expanding coverage. Advocates for expanded insurance reimbursement highlight IAHA’s cost-effectiveness in delaying surgeries such as TKA, reducing out-of-pocket expenses, and increasing accessibility. Developing cost-effective generic or biosimilar IAHA products and simplifying dosing regimens can reduce manufacturing and treatment costs and ensure competitive pricing while maintaining efficacy and safety. IAHA combined with PRP can effectively treat osteoarthritis (OA). IAHA offers pain relief and functional improvement for 3–6 months by enhancing joint lubrication and reducing inflammation, making it suitable for immediate symptom management, particularly in cases of moderate OA. PRP provides long-term benefits, including cartilage repair and anti-inflammatory effects, supporting sustained symptom relief and potential disease modification. PRP effects manifest gradually but last longer than six months. Combining IAHA’s rapid relief with PRP’s lasting benefits creates a balanced therapeutic approach [1,31,91,113]. IAHA is cost-effective in delaying surgical interventions such as TKA, especially in patients with moderate OA [18,55,58,92,178,179]. Reducing pain and improving joint function help postpone invasive procedures such as TJA, which is crucial for younger or active patients wanting to maintain mobility before surgery. Studies have shown that IAHA reduces healthcare costs by decreasing reliance on expensive interventions, such as joint replacement and long-term NSAIDs or corticosteroids, which carry systemic risk. IAHA’s ability of IAHA to prolong non-surgical treatment while improving quality of life underscores its value in multimodal OA care and economic benefits [1,32,80,91,113,132,179].

The limitations of the studies presented in this review include the fact that they cover a wide range of topics, including clinical guidelines and recommendations, IAHA efficacy, and alternative therapies, which may weaken the focus on specific aspects, such as the role of IAHA in multimodal approaches. The review is based on the available literature, in which many studies have been conducted in the short-to-medium term, with insufficient analysis of IAHA’s long-term efficacy and disease-modifying potential. Differences in the quality and methodology of the included studies led to challenges in synthesizing consistent conclusions, particularly regarding the comparative effectiveness of PRP and corticosteroids. Barely rudimentary cost-effectiveness analyses have been presented, and there has been limited discussion on how geographic differences in healthcare systems affect IAHA adoption. When discussing differences in clinical guidelines, the reasons for these discrepancies are not detailed, nor are there any suggestions for possible solutions to harmonization.

Clinical trials have endorsed IAHA for pain relief, functional improvement, and safety in specific subgroups of patients with OA. However, guidelines such as those from AAOS remain skeptical owing to stringent efficacy thresholds, variable evidence, and real-world barriers, such as cost and placebo effects. Harmonizing clinical trial standards, patient-specific recommendations, and long-term data is crucial for refining IAHA’s role of IAHA in OA treatment. Methodological inconsistencies in meta-analyses hinder the comparability of results and clinical translation. Standardized study designs, consistent outcome measures, and stratified analyses can enhance evidence reliability and clinical guidance. Incorporating IAHA into personalized regimens leverages synergistic effects, tailors treatment to individual needs, optimizes symptom relief, delays disease progression, and improves the quality of life of patients with OA. Tiered pricing, broader insurance coverage, and cost-effective alternatives could make IAHA more accessible, enhancing global OA treatment. Positioning IAHA as a mid-term solution complements PRP’s long-term potential, creating a staged or combined therapeutic approach for multimodal OA treatment. IAHA is a practical and economically viable strategy for managing OA symptoms and delaying surgery, particularly with conservative therapy. PRP’s long-term benefits and mechanical advantages make it a promising option for the comprehensive joint care of patients with OA.

Future research on IAHA should prioritize standardized, high-quality, multicenter RCTs with uniform protocols to assess its long-term efficacy and safety. Patient selection criteria (e.g., OA stage and biomarkers) should be standardized to identify subgroups most likely to benefit. Consistent outcome measures (e.g., WOMAC and VAS) should be used, and alternative therapies such as PRP and corticosteroids should be directly compared. Advanced formulations such as IAHA hybrids have been studied for their extended efficacy and chondroprotective potential. Evaluations of combination therapies, such as IAHA with PRP, for synergistic effects on symptom relief and joint protection should be conducted. Guidelines should be globally harmonized through international collaboration (e.g., OARSI, ESCEO, AAOS) to standardize recommendations and reduce disparities. Real-world evidence and cost-effectiveness analyses should be included in global guidelines to eliminate healthcare disparities. The focus should be placed on personalized medicine by developing biomarker-based tools to predict patient response, offer targeted treatments, and explore IAHA’s role in multimodal strategies combining lifestyle interventions with drug therapies.

## Figures and Tables

**Table 1:** A clear framework for interpreting the results’ variability and trends.

Parameter	Results/Details
Total Number of Studies	30 (systematic reviews, and meta-analyses)
Study Population	Approximate age: 62.4 years (±6.3)
	Female: 57%, Male: 43%
OA Severity	Early OA: 30% of studies
	Moderate OA: 50%
	Advanced OA: 20%
IAHA Formulations	High molecular weight: 40% of studies
	Low molecular weight: 35%
	Hybrid complexes: 25%
Comparators	Placebo: 40%
	Corticosteroids: 30%
	PRP: 20%
	NSAIDs: 10%
Outcomes Measured	Pain: WOMAC, VAS in 90% of studies
	Function: WOMAC Function in 80%
	Safety in 70%
Follow-Up Duration	Short-term (<6 months): 70%
	Long-term (≥6 months): 30%
Adverse Events Reported	Injection site pain/swelling: 10–15%
	Systemic reactions: <5%
Combination Therapies	IAHA + PRP: 15% of studies
	IAHA + corticosteroids: 10%
Efficacy Effect Sizes	Pain relief (WOMAC, VAS): Effect size 0.30–0.40 vs. placebo
	Functional improvement similar
Heterogeneity (I^2^)	Pain outcomes: Moderate heterogeneity (I^2^ = 50–60%)
	Functional improvement: High (I^2^ > 75%)
Cost-Effectiveness	IAHA delays surgical interventions
	Incremental cost-effectiveness ratios (ICERs) are reported sporadically.

**Table 2:** AMSTAR 2 results.

Study	PICO Definition	Comprehensive Search	Exclusions Justified	Risk of Bias	Meta-Analytic Methods	Impact of Bias	Heterogeneity	Funding	AMSTAR 2 Quality
Jevsevar et al. (2015) [29]	Clear	Comprehensive	Partially reported	Addressed with meta-regression	Robust	Discussed partially	High	Not disclosed	Moderate
Vincent et al. (2019) [33]	Clear	Comprehensive	Not discussed	Addressed partially	Robust statistical methods	Not explicitly addressed	Discussed partially	Not disclosed	Moderate
Chavda et al. (2022) [21]	Clear	Comprehensive	Not discussed	Addressed partially (PRISMA guidelines)	Systematic, robust	Not discussed	Addressed partially	Not disclosed	Moderate
Altman et al. (2016) [30]	Clear	Extensive	Not explicitly stated	Addressed partially (molecular weight subgroups)	Robust (meta-analysis)	Partially addressed	Discussed partially	Not disclosed	Moderate
Peck et al. (2021) [23]	Well-defined	Thorough	Not discussed	Partially addressed (safety limitations noted)	Appropriate statistical methods	Not explicitly discussed	Acknowledged	Not disclosed	High
Bannuru et al. (2015) [67]	Explicit	Comprehensive	Not explicitly mentioned	Addressed (Bayesian models used)	Robust network meta-analysis	Not explicitly discussed	Limited discussion	Not disclosed	High
Zhang et al. (2022) [52]	Explicit	Adequate	Not explicitly mentioned	Not adequately addressed	Robust (dual-network meta-analysis)	Addressed partially	Limited discussion	Not disclosed	High
Altman et al. (2015) [68]	Well-defined	Thorough	Partially addressed	Addressed with methodological quality tools	Systematic review	Not explicitly discussed	Partially addressed	Not disclosed	High
Bannuru et al. (2019) [69]	Explicit	Comprehensive	Not explicitly discussed	Addressed partially	Systematic guideline development	Partially addressed	Mentioned in passing	Not disclosed	Moderate
Honvo et al. (2019) [70]	Well-defined	Thorough	Partially discussed	Addressed with meta-analyses	Systematic, appropriate	Not explicitly addressed	Limited heterogeneity	Not disclosed	High
Webner et al. (2021) [71]	Clear	Comprehensive	Not discussed	Partially addressed	Systematic but no meta-analysis	Not addressed	Not addressed	Not disclosed	Moderate
Acuña et al. (2020) [72]	Explicit	Comprehensive	Not explicitly stated	Addressed partially	Systematic narrative synthesis	Not explicitly addressed	Acknowledged	Not disclosed	Moderate
Ali et al. (2021) [73]	Clear	Extensive	Not explicitly reported	Addressed partially	Statistical meta-analysis	Not explicitly addressed	Addressed partially	Not disclosed	Moderate
Anil et al. (2021) [74]	Explicit	Comprehensive	Not explicitly discussed	Addressed partially	Robust frequentist network meta-analysis	Not explicitly addressed	Partially discussed	Not disclosed	High
Concoff et al. (2019) [75]	Clear	Comprehensive	Partially discussed	Addressed partially	Systematic with mean differences	Partially addressed	Acknowledged	Not disclosed	High
Familiari et al. [37]	Explicit	Thorough	Not explicitly stated	Partially addressed	Meta-analysis (PEDro scale)	Not explicitly addressed	Partially addressed	Not disclosed	Moderate
Ferkel et al. (2023) [76]	Clear	Comprehensive	Not explicitly discussed	Partially addressed (subgroup analyses)	Statistical synthesis (basic science + clinical outcomes)	Addressed partially	Mentioned briefly	Not disclosed	High
Ferreira de et al. (2020) [77]	Explicit	Extensive	Not discussed	Addressed with AMSTAR 2 tools	Not applicable (protocol)	Addressed partially	Mentioned briefly	Not disclosed	High
Leite et al. (2018) [78]	Well-defined	Comprehensive	Not explicitly mentioned	Addressed partially (Cochrane RoB2)	Statistical synthesis (RCTs)	Addressed partially	Mentioned briefly	Not disclosed	Moderate
Mao et al. (2023) [79]	Explicit	Comprehensive	Not discussed	Addressed partially	Meta-analysis performed	Partially addressed	Discussed minimally	Not disclosed	High
Migliore et al. (2021) [80]	Clear	Thorough	Partially reported	Addressed with Delphi surveys	Narrative synthesis	Addressed briefly	Partially addressed	Not disclosed	High
Miller et al. (2020) [81]	Explicit	Comprehensive	Not explicitly stated	Addressed partially	Robust statistical methods	Not explicitly discussed	Addressed partially	Not disclosed	High
Patel et al. (2024) [82]	Clear	Comprehensive	Not explicitly discussed	Addressed partially (risk ratios)	Meta-analysis	Not explicitly discussed	Addressed partially	Not disclosed	Moderate
Riley et al. (2019) [83]	Clear	Extensive	Not explicitly discussed	Partially addressed	Meta-analysis (random-effects)	Partially addressed	Addressed partially	Not disclosed	Moderate
Singh et al. (2022) [84]	Explicit	Adequate	Not explicitly stated	Partially addressed (RCTs assessed)	Robust frequentist meta-analysis	Partially addressed	Addressed partially	Not disclosed	High
Wu et al. (2021) [85]	Well-defined	Comprehensive	Partially addressed	Addressed partially	Meta-analysis via RevMan	Not explicitly addressed	Discussed partially	Not disclosed	High
Xing et al. (2016) [65]	Clear	Comprehensive	Not explicitly discussed	Addressed with Jadad and AMSTAR	Best evidence synthesis	Addressed partially	Mentioned minimally	Not disclosed	High
Xing et al. (2017) [86]	Explicit	Thorough	Partially addressed	Addressed via ROBIS	Not applicable (synthesis of bias)	Discussed partially	Not applicable	Not disclosed	High
Aw et al. (2021) [87]	Explicit	Comprehensive	Not explicitly stated	Partially addressed (subgroup analyses)	Meta-analysis (pooled VAS, WOMAC)	Not explicitly addressed	Addressed partially	Not disclosed	High
Tan et al. (2021) [88]	Explicit	Comprehensive	Partially addressed	Partially addressed (subgroup analyses)	Meta-analysis performed	Not explicitly discussed	Discussed partially	Not disclosed	High
Zhang et al. (2022) [52]	Explicit	Comprehensive	Partially addressed	Partially addressed (subgroup analyses)	Meta-analysis performed	Not explicitly discussed	Discussed partially	Not disclosed	High

**Table 3:** The impact of IAHA, IA-CS, and PRP therapies on their efficacy, safety, and cost-effectiveness.

Therapy	Efficacy	Safety	Cost-Effectiveness
IAHA	Medium-term, gradual relief	Favorable, rare side effects	Cost-effective in early/moderate OA
IA-CS	Short-term, fast relief	Risk of systemic effects	Low-cost but repetitive use
PRP	Long-term, regenerative potential	The variable needs further validation	High-cost, experimental

**Table 4:** Summary of the recommendations of selected associations and how they have changed during the past decade.

Organization	Period 2014–2021	Period 2022–2024
AAOS	Against	Against
Swiss Society and German Orthopaedic Society	Cautious	Cautious
ACR	Conditional	Conditional
AMSSM	Delaying surgery in active patients	Delaying surgery in active patients
ICRS	Endorses early use to delay surgery	Endorses early use to delay surgery
EULAR	Personalized care	Personalized care
Brazilian Society	Recommends UHMW	Recommends UHMW
Spanish Society	Recommends VS	Recommends VS
ESCEO and ISIAT and SIOT	Supportive	Supportive
Korean Guidelines	Supports conditional use	Supports conditional use
SARAA	Supports relief and delay	Supports relief and delay
Indian Rheumatology	UHMW for sustained relief	UHMW for sustained relief
OARSI	Uncertain	Uncertain
NICE (UK)	Uncertain	Against

## Data Availability

No new data were created for this study.
